# Lysine-specific demethylase 1 inhibitors prevent teratoma development from human induced pluripotent stem cells

**DOI:** 10.18632/oncotarget.24030

**Published:** 2018-01-08

**Authors:** Naoki Osada, Jiro Kikuchi, Takashi Umehara, Shin Sato, Masashi Urabe, Tomoyuki Abe, Nakanobu Hayashi, Masahiko Sugitani, Yutaka Hanazono, Yusuke Furukawa

**Affiliations:** ^1^ Division of Stem Cell Regulation, Center for Molecular Medicine, Jichi Medical University, Shimotsuke, Tochigi 329-0498, Japan; ^2^ Epigenetic Drug Discovery Unit, RIKEN Center for Life Science Technologies, Yokohama, Kanagawa 230-0045, Japan; ^3^ Division of Cell and Gene Therapy, Center for Molecular Medicine, Jichi Medical University, Shimotsuke, Tochigi, 329-0498, Japan; ^4^ Division of Regenerative Medicine, Center for Molecular Medicine, Jichi Medical University, Shimotsuke, Tochigi 329-0498, Japan; ^5^ Gene Try Company, Itabashi, Tokyo 173-8610, Japan; ^6^ Department of Pathology, Nihon University School of Medicine, Itabashi, Tokyo 173-8610, Japan

**Keywords:** LSD1, small molecule inhibitor, teratoma, iPS, regenerative medicine

## Abstract

Human induced pluripotent stem cells (hiPSCs) are creating great expectations for regenerative medicine. However, safety strategies must be put in place to guard against teratoma formation after transplantation of hiPSC-derived cells into patients. Recent studies indicate that epigenetic regulators act at the initial step of tumorigenesis. Using gain-of-function and loss-of-function approaches, we show here that the expression and function of lysine-specific demethylase 1 (LSD1) are tightly regulated in hiPSCs, and their deregulation underlies the development of teratomas. Consistent with these results, we demonstrate that an LSD1 inhibitor, S2157, prevented teratoma formation from hiPSCs transplanted into immunodeficient mice. This novel action of LSD1 and the effects of its inhibition potentially allow for the development of new clinical applications and therapeutic strategies using hiPSCs.

## INTRODUCTION

Human induced pluripotential stem cells (hiPSCs) can be generated from adult somatic cells simply by introduction of reprogramming factors [1]. This enables the use of patient-specific hiPSCs, which presents fewer ethical concerns than use of embryonic stem cells (ESCs) and has a lower risk of rejection [2–4]. Nonetheless, the clinical application of hiPSCs still entails some important risks, one of which is the oncogenic transformation of residual undifferentiated hiPSCs [5, 6].

Recently, a first in-human clinical trial was conducted using autologous hiPSC-derived cells to treat age-related macular degeneration [7]. In this study, iPSCs were generated from skin fibroblasts of two patients and differentiated into retinal pigment epithelial (RPE) cells. Whole genome sequencing revealed no genomic aberrations with tumorigenic potential in the hiPSC-derived RPE cells from the first patient, but copy number alterations were detected in those from the second patient. In the first patient, transplanted cells remained intact without tumor development one year after transplantation. The second patient did not undergo transplantation, although the genetic change was not considered directly tumorigenic according to published literature [5, 6].

Human iPSCs reportedly develop teratomas more efficiently and faster than human ESCs, regardless of injection site [8], and that as few as 100 iPSCs or ESCs are sufficient to produce a teratoma [9, 10]. Therefore, safety strategies must be put in place to guard against teratoma formation after transplantation of hiPSC-derived cells. hiPSC genetic modification methods, such as the introduction of suicide genes, have several limitations in terms of specificity, throughput, efficacy, and safety [11]. Therefore, other strategies based on different mechanisms are needed.

Recent studies revealed that epigenetic deregulation plays a fundamental role in oncogenesis [12]. More importantly, epigenetic abnormalities generally act as initiating mutations, which transform normal stem cells into cancer stem cells at the initial step of oncogenesis. In addition, Ohnishi et al. [13] showed that hiPSC-derived teratomas do not exhibit genetic abnormalities. These findings suggest that epigenetic alterations act as drivers to promote teratoma formation, but further investigation is necessary.

In the present study, we used gain-of-function and loss-of-function approaches to determine that expression of lysine-specific demethylase 1 (LSD1/KDM1A) is elevated in hiPSC-derived teratomas and that LSD1 deregulation underlies teratoma development. In addition, we demonstrate a novel strategy for preventing teratoma formation using LSD1 inhibitors after transplantation, which could increase the safety of clinical applications of hiPSCs.

## RESULTS

### LSD1 is minimally expressed in hiPSCs, but strongly expressed in hiPSC-derived teratoma

**Figure 1:**
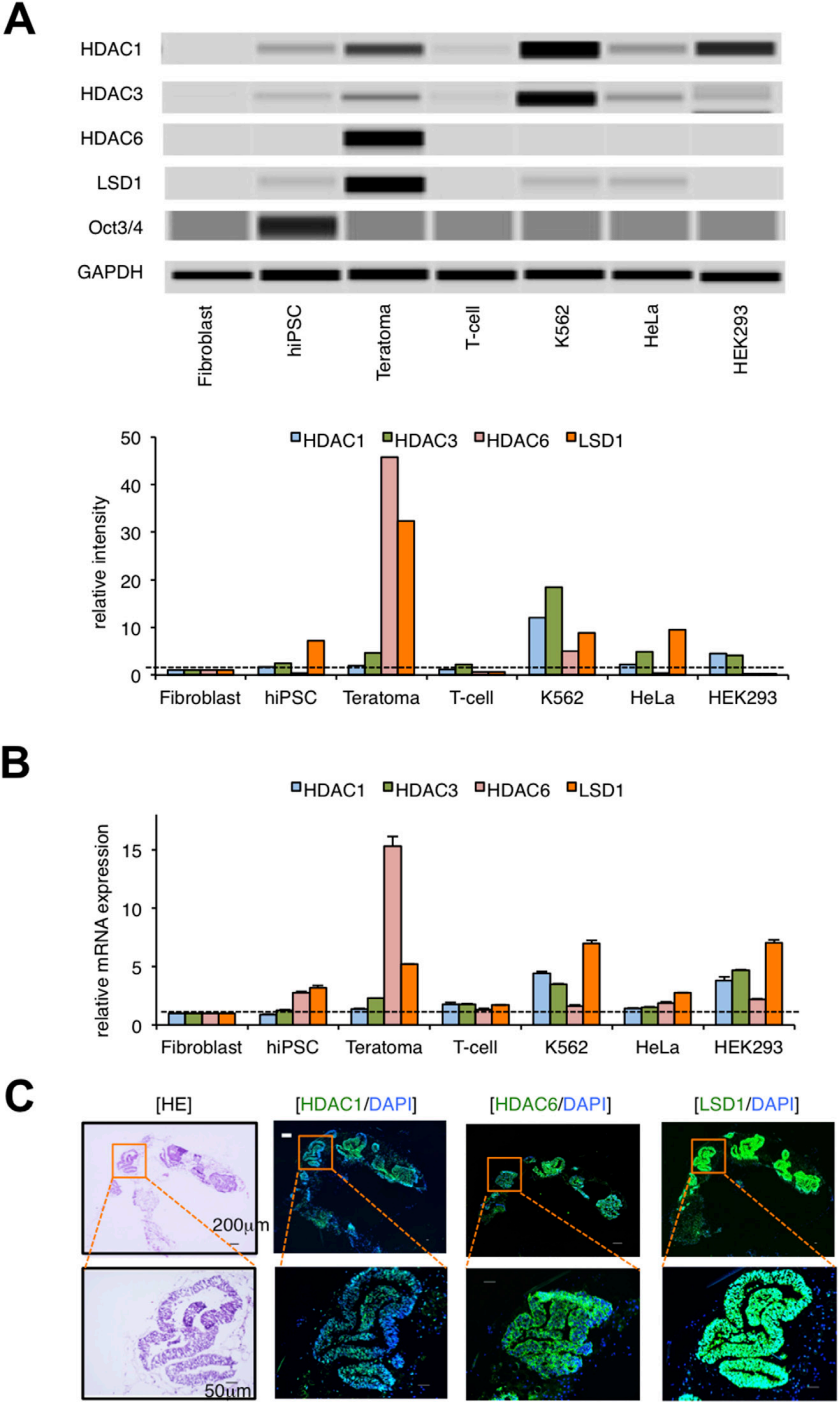
LSD1 is minimally expressed in hiPSCs but strongly expressed in hiPSC-derived teratoma **(A)** We isolated whole cell lysates from normal human fibroblasts, hiPSC line 201B, 201B-derived teratoma, normal human T-lymphocytes, and cancer cell lines (K562, HeLa, and HEK293) for immunoblot analyses to determine the expression of HDAC1, HDAC3, HDAC6, LSD1, Oct3/4, and GAPDH (internal control) (upper panel). The signal intensities of each band were quantified, normalized to those of the corresponding GAPDH, and shown as relative values setting fibroblast at 1.0 (lower panel). Immunoblotting was carried out using the Simple Western System Wes. **(B)** We isolated total cellular RNAs and subjected them to qPCR to evaluate the expression of *HDAC1, HDAC3, HDAC6*, and *LSD1* mRNA. Data were quantified by the 2^–ΔΔCt^ method using simultaneously amplified *GAPDH* as a reference and are shown as relative values setting fibroblast at 1.0. **(C)** Frozen continuous sections were prepared from the developed teratomas and subjected to hematoxylin-eosin (HE) and immunofluorescent chemical (IFC) staining. IFC specimens were stained with anti-HDAC1, anti-HDAC6, or anti-LSD1 antibodies, followed by staining with Alexa Fluor 488-conjugated anti-rabbit IgG (green). Nuclei were counterstained with DAPI (blue). Only merged images are shown. Scale bars indicate 200 μm (upper panels) and 50 μm (lower panels), respectively. Data shown are representative of multiple independent experiments.

To elucidate the epigenetic mechanisms of teratoma formation from hiPSCs, we compared the expression of epigenetic regulators in normal somatic cells, hiPSCs, hiPSC-derived teratoma, and cancer cell lines by immunoblot analysis. The examined molecules include major class I and II histone deacetylases (HDACs), histone demethylases LSD1, JMJD2A and JMJD2D, histone methyltransferases EZH1, EZH2 and G9a, and DNA methyltransferase DNMT1, most of which have been shown to be deregulated in cancer cells [12–14]. Among them, HDAC1, HDAC3, and LSD1 were barely expressed in hiPSCs and normal somatic cells (fibroblasts and T-lymphocytes), but they were strongly expressed in hiPSC-derived teratomas and cancer cell lines (K562, HeLa and HEK293) at the protein level (Figure 1A and Supplementary Figure 1). Notably, the expression of HDAC6 and LSD1 was much higher in teratomas than other normal and transformed cells. Real-time quantitative RT-PCR analyses confirmed the increased expression of LSD1 in teratomas comparable to those in cancer cell lines at the mRNA level (Figure 1B). Next, we performed immunofluorescent chemical (IFC) staining to investigate which types of cells express them in hiPSC-derived teratomas. Most hiPSC-derived differentiated cells expressed HDAC1, HDAC6, and LSD1 (Figure 1C and Supplementary Figure 2). These results suggest that the deregulated expression of HDAC1, HDAC6 and LSD1 may underlie the development of teratoma. To determine the most critical one(s) among them, we tested the inhibitory effects of the pan-HDAC inhibitor panobinostat, the HDAC6-specific inhibitor tubastatin A, and the proteasome inhibitor bortezomib, which exerts anti-tumor effects on teratoma formation via the down-regulation of class I HDACs [14, 15]. None of these compounds inhibited teratoma formation from hiPSCs in immunodeficient mice (Supplementary Figure 3 and data not shown). Taken together, LSD1, but not HDACs, appears to play a critical role in hiPSC-induced teratoma formation.

### LSD1 is strongly expressed in hiPSC-derived teratoma and its derivatives of all three germ layers

**Figure 2:**
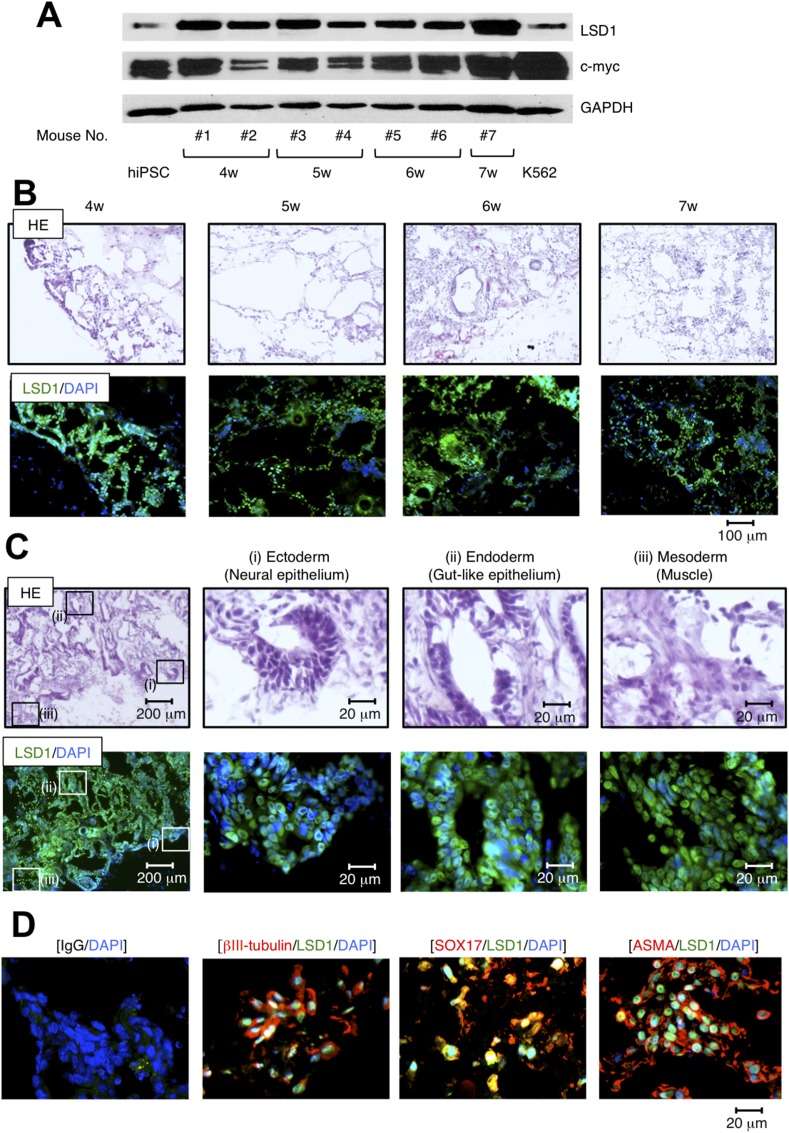
LSD1 is strongly expressed in derivatives of all three germ layers during teratoma formation and growth **(A)** We isolated whole cell lysates from ChiPS17-derived teratomas at the indicated time points and subjected them to immunoblot analyses to determine the expression of LSD1, c-Myc, and GAPDH (internal control). **(B)** Frozen continuous sections were prepared from teratomas at the indicated time points and subjected to HE and IFC staining. IFC specimens were stained with anti-LSD1 antibody, followed by staining with Alexa Fluor 488-conjugated anti-rabbit IgG (green). Nuclei were counterstained with DAPI (blue). Only merged images are shown. Scale bars indicate 100 μm. **(C)** Frozen continuous sections of teratomas were subjected to HE and IFC staining. IFC specimen was stained with anti-LSD1 antibody, followed by staining with Alexa Fluor 488-conjugated anti-rabbit IgG (green). Nuclei were counterstained with DAPI (blue). Only merged images are shown. Scale bars indicate 200 μm (left panels) and 20 μm, respectively. **(D)** Frozen sections of teratomas were subjected to immunofluorescent chemical (IFC) staining. IFC specimens were stained with antibodies against LSD1, ßIII-tubulin, SOX17 and ASMA, followed by staining with Alexa Fluor 488-conjugated anti-rabbit IgG (green) or Alexa Fluor 594-conjugated anti-mouse IgG (red). Nuclei were counterstained with DAPI (blue). Only merged images are shown. Scale bars indicate 20 μm. Data shown are representative of multiple independent experiments.

To confirm the role of LSD1 in teratoma formation, we determined the time-course of LSD1 expression in hiPSC-derived teratomas using samples obtained from transplanted mice every week for 4 weeks after inoculation. Immunoblot analyses revealed that the expression level of LSD1 was readily increased in teratomas compared with original iPSCs and that LSD1 was higher than that in K562 leukemia cells at any time point examined (Figure 2A). Previous studies have indicated that c-Myc plays a pivotal role in the tumorigenesis of hiPSCs [5], and its expression is epigenetically regulated by LSD1 in cancer cells [16]. Consistent with these previous findings, c-Myc was strongly expressed in teratomas and had a positive correlation with the abundance of LSD1 expression (Figure 2A). Hematoxylin-eosin (HE) and IFC staining of continuous sections confirmed the expression of LSD1 in most hiPSC-derived teratoma cells at all time points examined (Figure 2B and Supplementary Figure 4). These results suggest that LSD1-mediated epigenetic abnormalities act as an initiating event in hiPSC-induced teratoma formation.

Since hiPSCs are capable of pluripotent differentiation, we determined the expression of LSD1 in derivatives from the three germ layers in hiPSC-derived teratoma tissues. As shown in Figure 2C, HE staining confirmed formations of three different germ layers in the teratomas, such as neural epithelium (ectoderm), gut-like epithelium (endoderm), and muscle (mesoderm). IFC staining of continuous sections confirmed that LSD1 is robustly expressed in all three layers (Figure 2C and Supplementary Figure 4), along with the lineage-specific markers ßIII-tubulin, SOX17, and ɑ-smooth muscle actin (Figure 2D). These results suggest that LSD1 also plays a role in the differentiation and/or maintenance of hiPSC-induced teratomas.

### Genetic modifications modulates LSD1 expression and teratoma formation from iPSCs

**Figure 3:**
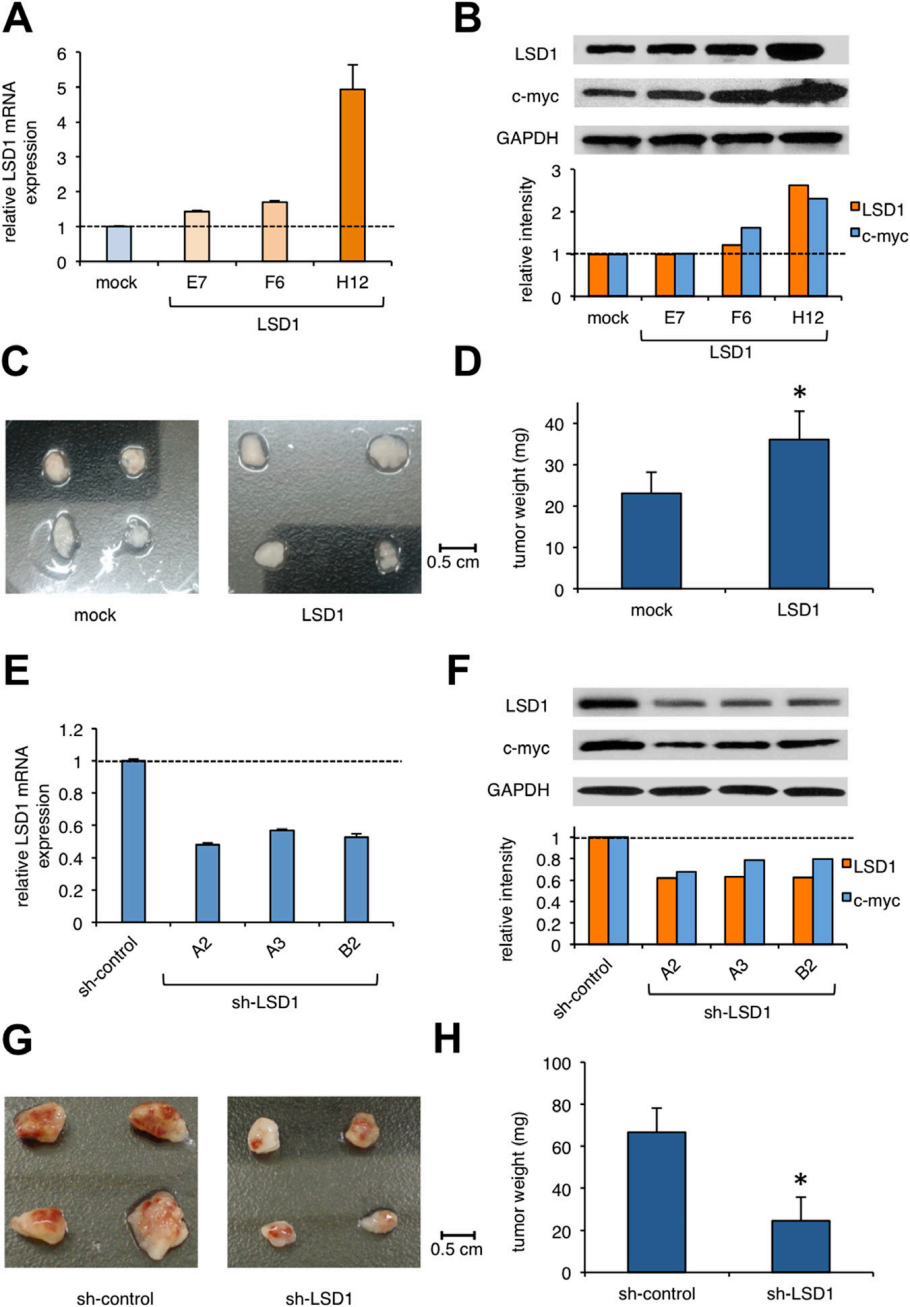
The effect of LSD1 overexpression or knockdown on teratoma formation **(A)** We established sublines from hiPSC line ChiPS17 by transducing with an empty plasmid (mock) or expression vector for LSD1 (E7, F6, and H12). Total cellular RNA was isolated from the sublines and subjected to qPCR for the expression of LSD1. Data were quantified by the 2^-ΔΔCt^ method using simultaneously amplified GAPDH as a reference and shown as relative values against mock-transfected controls. **(B)** We isolated whole cell lysates from hiPSC sublines for immunoblot analyses on the expression of LSD1, c-Myc, and GAPDH (internal control) (upper panel). The signal intensities of each band were quantified, normalized to those of the corresponding GAPDH, and shown as relative values with mock at 1.0 (lower panel). **(C** and **D)** NOD/SCID mice were inoculated subcutaneously with 2 × 10^6^ cells of ChiPS17-mock or ChiPS17-LSD1-H12 into the right thigh. Representative photographs of the teratomas on day 22 (original magnification: ×2). The y-axis shows the average tumor weight of the teratomas. The means ± S.D. (bars) are shown. *Asterisk* indicate *P* <0.05 by Student’s *t* test. **(E)** We obtained ChiPS17 sublines transduced with sh-RNA against against *LSD1* (A2, A3, and B3) and ineffective control (sh-control). Total cellular RNA was isolated from the sublines and subjected to qPCR for the expression of LSD1. Data were quantified by the 2^-ΔΔCt^ method using simultaneously amplified GAPDH as a reference and shown as relative values against sh-controls. **(F)** We isolated whole cell lysates from hiPSC sublines for immunoblot analyses to determine the expression of LSD1, c-myc, and GAPDH (internal control) (upper panel). The signal intensities of each band were quantified, normalized to those of the corresponding GAPDH, and shown as relative values with mock at 1.0 (lower panel). **(G** and **H)** NOD/SCID mice were inoculated subcutaneously with 2 × 10^6^ cells of ChiPS17-sh-control or ChiPS17-sh-LSD1-A2 into the right thigh. Representative photographs of teratomas on day 35 (original magnification: ×2). The y-axis shows the average tumor weight of the developed teratomas. The means ± S.D. (bars) are shown. *Asterisks* indicate *P* <0.05 by Student’s *t* test.

The above findings suggest that LSD1 is repressed in hiPSCs, and its overexpression predisposes them to the development of teratomas. To obtain compelling evidence to support this notion, we established LSD1-overexpressing hiPS sublines by lentiviral transduction of *LSD1* cDNA (Figure 3A) and confirmed the overexpression of LSD1 and c-Myc in the subline H12 (Figure 3B). No obvious difference was noted in the morphology or the expression levels of pluripotent markers, including Oct3/4, Sox2, and KLF4 between mock- and LSD1-transduced hiPSCs (data not shown). These sublines could be passaged in a routine manner, similar to untransduced hiPSCs. LSD1 overexpression itself did not affect the proliferative potential or viability of hiPSCs *in vitro* (data not shown). Upon transplantation into immunodeficient mice, however, the H12 subline grew faster than the mock-transfected control. On day 22 of transplantation, tumors weighed significantly more in teratomas derived from LSD1-overexpressing hiPSCs (Figure 3C/3D). HE staining showed that teratomas contained derivatives of the three germ layers without any obvious difference between mock- and LSD1-transduced hiPSCs (Supplementary Figure 5), indicating the retained capacity of pluripotent differentiation in the H12 subline.

Next, we performed knockdown experiments to confirm the role of LSD1 in teratoma formation. We established hiPS sublines lentivirally transduced with short-hairpin RNA against LSD1 (sh-LSD1) and an ineffective control (sh-control). We used the subline A2, in which the expressions of LSD1 and c-Myc were obviously down-regulated relative to those in the control (Figure 3E/3F). LSD1 silencing itself did not affect the proliferative potential or viability of hiPSCs (data not shown). We inoculated these sublines into immunodeficient mice and compared the growth *in vivo*. On day 35 of transplantation, tumor weights were significantly lower for teratomas derived from LSD1-knockdown hiPSCs (Figure 3G/3H and Supplementary Figure 6). These results suggest that LSD1 has a critical role in teratoma formation, growth and maintenance, and thus, could be a therapeutic target for preventing teratoma formation from hiPSCs.

### Novel LSD1 inhibitors inhibit proliferation and differentiation of hiPSCs *in vitro*

**Figure 4:**
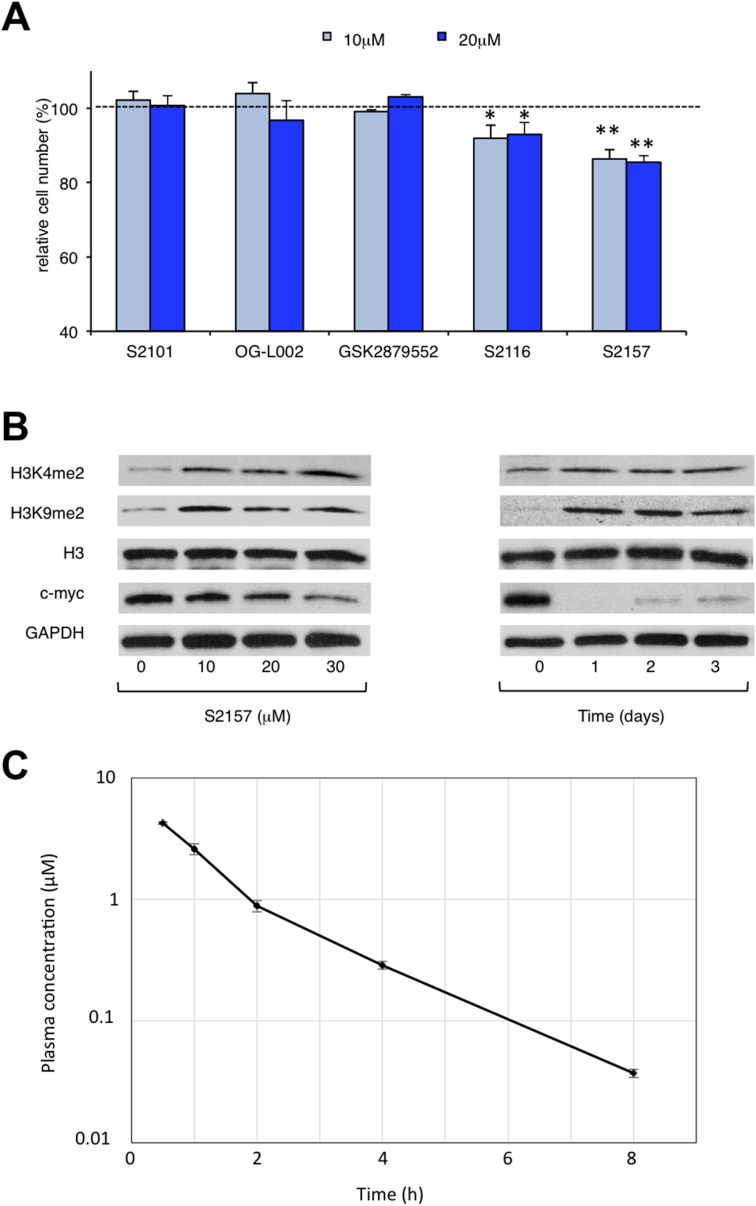
Novel LSD1 inhibitors prevent the proliferation and differentiation of hiPSCs *in vitro* **(A)** Cell proliferation was measured by MTT assays after ChiPS17 cells were cultured in the differentiation medium (DMEM supplemented with 10% FCS and 10 μM Y27632) without or with S2101, OG-L002, GSK2879552, S2116 or S2157 at the indicated doses for 72 h. Absorbance at 450 nm was measured with a microplate reader and expressed as a percentage of the value of corresponding untreated cells. The means ± S.D. (bars) are shown. ^**^P <0.05 *vs* S2101, OG-L002, GSK2879552 and S2116; ^*^P <0.05 *vs* S2101, OG-L002 and GSK2879552 determined by one-way ANOVA with Tukey’s multiple comparison test (n = 3–6). **(B)** Left panel: ChiPS17 cells were cultured in the differentiation medium without or with S2157 at the indicated doses for 24 h. Whole cell lysates were subjected to immunoblotting for the expression of H3K4me2, H3K9me2, histone H3, c-Myc, and GAPDH (internal control). Right panel: ChiPS17 cells were cultured with 30 μM S2157 for up to 3 days. Whole cell lysates were prepared at the given time points and subjected to immunoblotting as described above. **(C)** Pharmacokinetic profile of S2157 in plasma following a single administration of S2157 (50 mg/kg; intraperitoneal) to mice. The means ± S.D. (bars) are shown (*n* = 3).

The above findings prompted us to investigate whether the administration of a small molecule inhibitor against LSD1 could prevent teratoma formation from hiPSCs. LSD1 belongs to the flavin adenine dinucleotide (FAD)-dependent amine oxidase family. A prototype LSD1 inhibitor, tranylcypromine (TCP), acts through competitive binding to FAD and inhibits two major isoforms of monoamine oxidases MAO-A and MAO-B with lower Ki values than LSD1 [17]. A series of TCP derivatives with increased specificity to LSD1 have been developed and used in cancer biology to develop anti-cancer agents [18, 19]. For example, one of these compounds, S2101, reduced the viability of glioblastoma stem cells [20, 21]. We examined the effects of S2101 and two other TCP derivatives (OG-L002 and GSK2879552) on the proliferation of hiPSCs under differentiation-inducing conditions *in vitro*. Unfortunately, these compounds only affected the growth of hiPSCs at concentrations unachievable *in vivo* (> 40 μM) (data not shown). Therefore, we tested several *N*-alkylated derivatives of S2101 with lower Ki values and higher specificity to LSD1 than LSD2 and MAO-A/B, such as S2116 and S2157 (Table 1). We found that S2157 considerably suppressed the proliferation of hiPSCs at 10 μM, whereas S2116 had only a moderate, albeit still significant, inhibitory effect (Figure 4A). We selected S2157 for further experiments because of the higher efficacy. As shown in Figure 4B, S2157 readily inhibited the histone demethylase activity of LSD1 at 10 μM, as evidenced by the increased di-methylation levels at lysine 4 and lysine 9 of histone H3 (H3K4me2 and H3K9me2), which coincided with down-regulation of c-Myc expression in a dose- and time-dependent manner in hiPSCs. A pharmacokinetic analysis of S2157 in mouse plasma (Figure 4C) showed relatively rapid metabolism with C_max_ of 3.6 μM (T_max_ = 0.5 h) and an AUC of 5.5 μM·h after a single intraperitoneal dose of 50 mg/kg. Taken together, these results suggest that S2157 could interfere with the proliferation and differentiation of hiPSCs via the inhibition of LSD1 activity *in vitro*. Therefore, we examined the *in vivo* efficacy of S2157 at preventing teratoma formation from hiPSCs.

**Table 1:** IC_50_ values of LSD1 inhibitors

Compound	Chemical structure	LSD1 (μM)	LSD2 (μM)	MAO-A (μM)	MAO-B (μM)
S2101	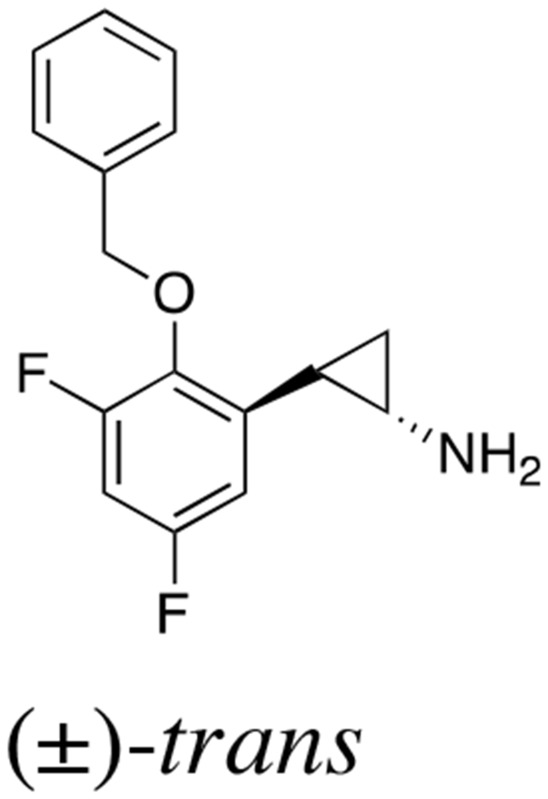	1.7 ± 0.015	380 ± 41	69 ± 2.4	35 ± 3.0
S2116	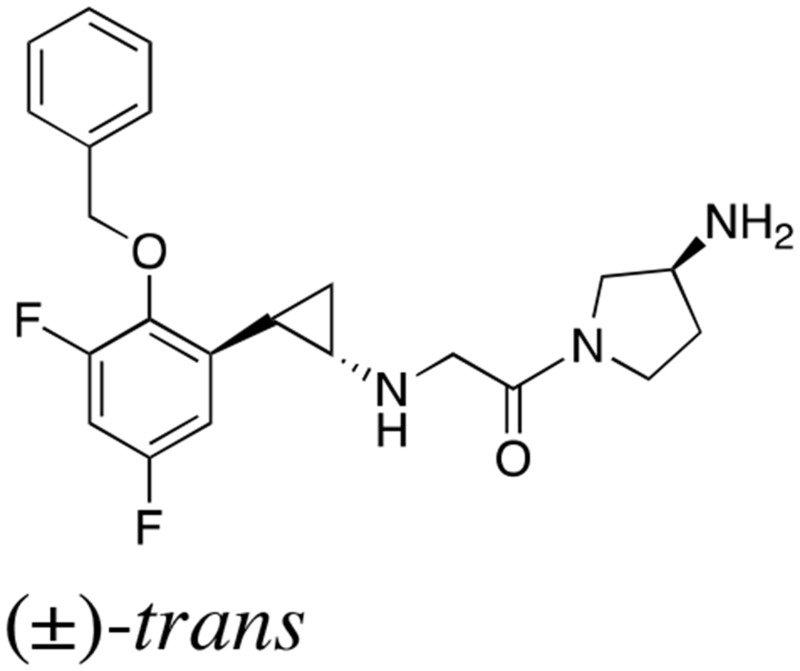	0.74 ± 0.070	> 500	> 500	> 500
S2157	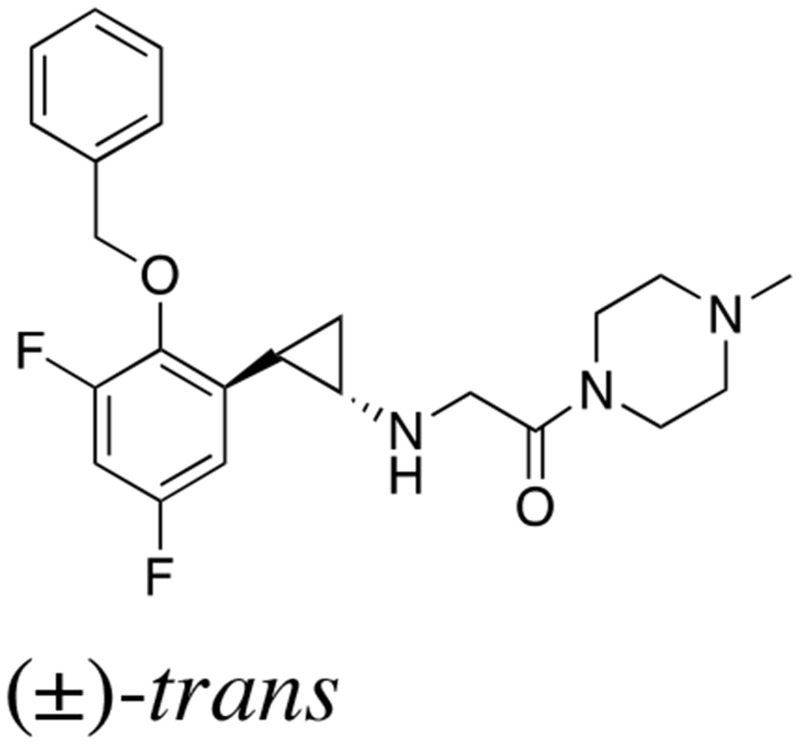	0.89 ± 0.014	450 ± 13	380 ± 14	220 ± 35

### S2157, a novel LSD1 inhibitor, prevents teratoma formation from hiPSCs

**Figure 5:**
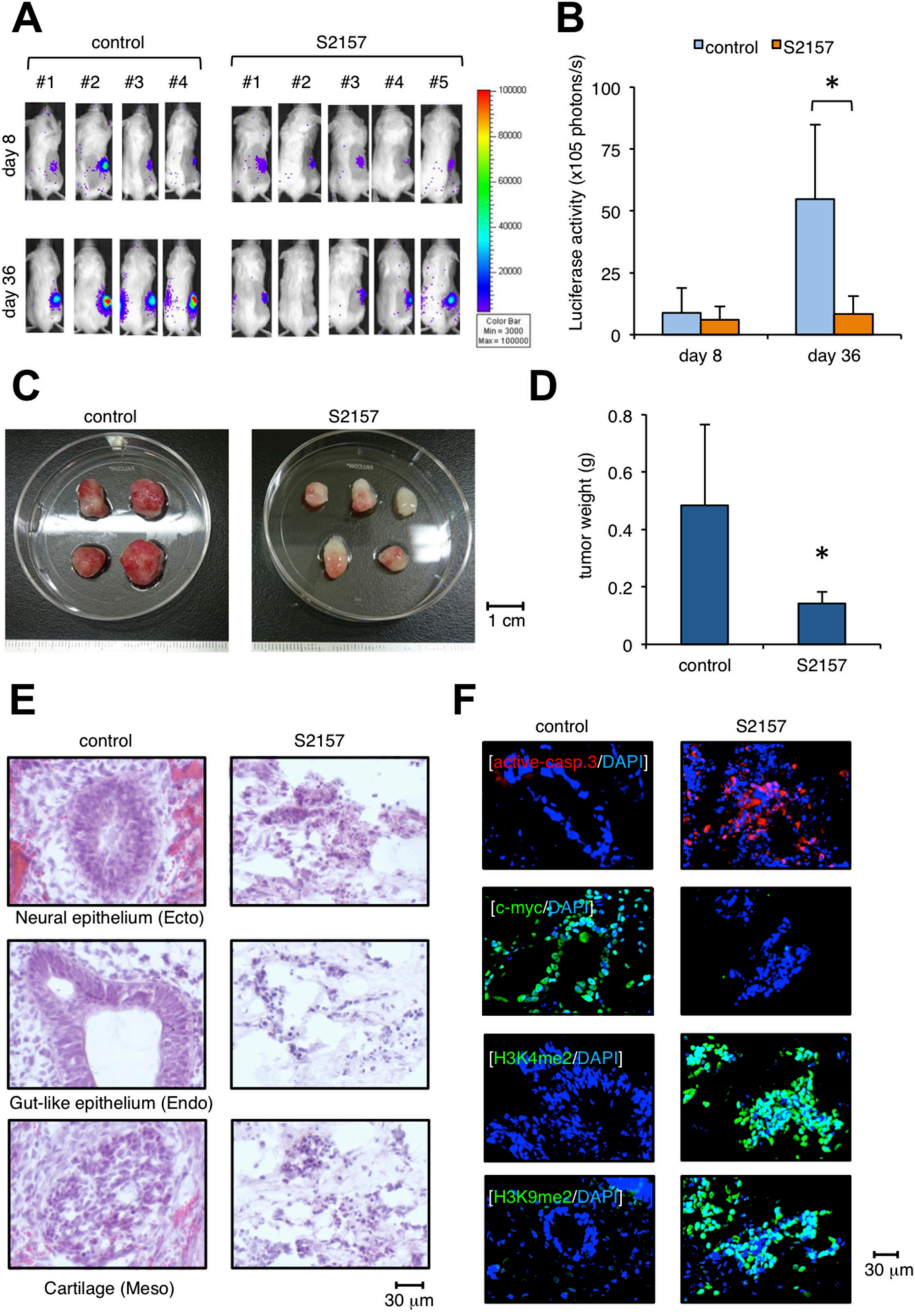
Administration of the LSD1 inhibitor S2157 prevents teratoma formation from hiPSCs **(A)** We subcutaneously inoculated 2 × 10^6^ luciferase-expressing ChiPS17-Luc cells in the right thigh of NOD/SCID mice. Immediately after transplantation, mice were intraperitoneally administered 50 mg/kg S2157 (n=5) or vehicle (0.9% NaCl) (n=4) six times per week for four weeks. Representative photographs of mice are shown (original magnification: ×2). **(B)** Quantitative data of *in vivo* bioluminescence imaging on day 8 and day 40 expressed in units of photons (photons/s). *Asterisk* indicates *P* <0.05 by Student’s *t* test. **(C)** Representative photographs of teratomas on day 40 (original magnification: ×2). **(D)** The y-axis shows the average tumor weight of the developed teratomas. The means ± S.D. (bars) are shown. *Asterisk* indicates *P* <0.05 by Student’s *t* test. **(E)** HE staining of frozen sections prepared from the developed teratomas. **(F)** IFC staining of frozen sections prepared from the developed teratomas. The specimens were stained with PE-conjugated anti-active caspase-3 (red), anti-c-myc, anti-H3K4me2, or anti-H3K9me2 antibody, followed by staining with Alexa Fluor 488-conjugated anti-rabbit IgG (green). Nuclei were counterstained with DAPI (blue). Only merged images are shown. Scale bars indicate 30 μm. Data shown are representative of multiple independent experiments.

To investigate the preventive effect of S2157 on teratoma formation, we established a luciferase-expressing hiPSC subline and inoculated it into immunodeficient mice. Immediately after transplantation, either S2157 (50 mg/kg) or vehicle (3% DMSO in 0.9% NaCl) was intraperitoneally administered six times per week for four weeks (n=4-5 in each group). We compared tumor sizes between vehicle- and S2157-treated groups by *ex vivo* monitoring of luciferase activity on days 8 and 36. As shown in Figure 5A/5B, S2157 significantly retarded the growth of inoculated cells on day 36. In addition, tumor weight was significantly lower in the S2157-treated group than in the vehicle-control group (Figure 5C-5D). No obvious side effects, including body weight reduction, leukocytopenia, thrombocytopenia, and anemia, were associated with S2157 treatment (data not shown). HE staining confirmed the presence of derivatives of three germ layers in the vehicle-control group, whereas, massive cell apoptosis was observed in corresponding tissues in the S2157-treated group (Figure 5E). IFC staining detected caspase-3 activation and c-Myc down-regulation, which coincided with the increased abundance of H3K4me2 and H3K9me2 under treatment with S2157 (Figure 5F). This preventive effect was confirmed in mice transplanted with the other hiPSC cell line, 201B (Supplementary Figure 7). Taken together, these data demonstrate that S2157 has potent anti-teratomagenic ability via the inhibition of LSD1 activity and the induction of apoptosis.

## DISCUSSION

In the present study, we show that the expression and function of LSD1 are tightly regulated in hiPSCs, and their dysregulation underlies teratoma development. We therefore developed a novel strategy to prevent teratoma formation after transplantation of hiPSC-derived cells using the LSD1 inhibitor S2157. This is the first report of therapeutic intervention against teratoma formation by targeting an epigenetic regulator with initiator function. Notably, because LSD1 is barely expressed in most terminally differentiated somatic cells, LSD1 inhibitors may selectively eradicate teratoma without detrimental effects to hiPSC-derived differentiated cells. The use of LSD1 inhibitors could thus increase the safety of the clinical applicability of hiPSCs.

LSD1 and HDAC respectively mediate repressive function via H3K4 demethylation and global histone deacetylation as components of the REST corepressor (CoREST) and nucleosome remodeling and deacetylase (NuRD) complex in hiPSCs [22]. The LSD1-NuRD complex decommissions enhancers of the pluripotency program during differentiation and is essential for complete shutdown of the ESC gene expression program. It is reasonable to speculate that hyperactivity of LSD1-containing complexes underlies the development of teratoma from pluripotent cells. Indeed, LSD1 inhibitors, but not HDAC inhibitors, were effective for preventing teratoma formation. Consistent with this finding, previous studies demonstrated that loss of HDAC1 and HDAC2 did not inhibit teratoma formation from ESCs [23]. Loss of LSD1 is embryonically lethal [24] with failure to differentiate into embryoid bodies [25, 26].

LSD1 bifunctionally modulates the enhancer/promoter functions of target genes via removing the mono- and di-methyl groups from H3K4 and H3K9 [27, 28]. During oncogenesis, LSD1 seems to act predominantly as a histone H3K9 demethylase to de-repress the transcription of oncogenes. For example, LSD1 overexpression increases the expression of HOXA family members, which act to generate pre-leukemic stem cells predisposing to the development of hematological malignancies [29]. LSD1 may act in a similar manner in hiPSCs by up-regulating the expression of c-myc and generates cancer stem cells for development of teratoma. In addition, Yoshihara et al. [30] reported that point mutations occurred preferentially in heterochromatic regions enriched for H3K9 trimethylation in both mouse and human iPSCs. LSD1 hyperactivation may induce the expression of mutated genes and/or conformational changes in these regions by erasing repressive H3K9 modifications. These findings strongly suggest the possibility that hiPSCs neither differentiate nor maintain the cancer stem cell state in the presence of LSD1 inhibitors, resulting in cell death.

## MATERIALS AND METHODS

### Cells and cell culture

The human iPS cell line 201B was provided by Dr. Shinya Yamanaka (Department of Life Science Frontiers, Center for iPS Cell Research and Application, Kyoto University, Kyoto, Japan). 201B cells were maintained in tissue culture plates coated with recombinant human truncated vitronectin (Invitrogen, Carlsbad, CA) in Essential 8 medium (Invitrogen) supplemented with 10 μM ROCK inhibitor Y27632 (Invitrogen). Cells were routinely passaged as small clumps using the EDTA method with the split ratio of 1:8 to 1:12 every 2 to 3 days after reaching 60% to 80% confluence [31]. The human iPSC line ChiPS17 was purchased from TaKaRa (Shiga, Japan) and cultured using Cellartis DEF-CS Culture System (TaKaRa) according to the manufacturer’s instructions. Human fibroblast BJ and cancer cell lines (K562, HeLa and HEK293) were purchased from the Health Science Research Resources Bank (Osaka, Japan) and maintained in RPMI1640 or DMEM medium (Sigma-Aldrich, St. Louis, MO) supplemented with 10% heat-inactivated fetal calf serum (FCS) (Sigma-Aldrich).

### Immunoblotting

We carried out immunoblotting using the Wes™ Simple Western System (Protein Simple, San Jose, CA) and specific antibodies against HDAC1-8 (Bethyl Laboratories Inc, Montgomery, TX), LSD1 (Cell Signaling Technology, Beverly, MA), Oct3/4 (Invitrogen), ßIII-tubulin, SOX17, ɑ-smooth muscle actin (Gene Tex Inc, Irvine, CA), di-methylated H3K4 (Active Motif, Carlsbad, CA), di-methylated H3K9 (Millipore), and GAPDH (Cell Signaling Technology).

### Immunofluorescence staining

Frozen sections of teratomas were prepared for immunostaining as described previously [32]. We used Alexa Fluor 488-conjugated anti-rabbit IgG and Alexa Fluor 594-conjugated anti-mouse IgG (Invitrogen) as secondary antibodies. Nuclei were counterstained with DAPI.

### Quantitative reverse transcription-polymerase chain reaction (qRT-PCR)

Total cellular RNA was isolated from 10 × 10^4^ cells using an RNeasy Kit (Qiagen, Valencia, CA, USA), reverse-transcribed into complementary DNA using ReverTra Ace and oligo(dT) primers (Toyobo, Tokyo, Japan), and subjected to quantitative real-time RT-PCR (qPCR). We used the Expression Assays (Hs01002741 for LSD1, Hs00606262 for HDAC1, Hs00187320 for HDAC3, Hs00997427 for HDAC6, Hs04260367 for Oct3/4, Hs00415716 for Sox2, and Hs01922876 for GAPDH) and a TaqMan Fast Universal PCR Master Mix.

### Construction and production of lentiviral expression vectors

We used the lentiviral vector CSII-CMV-MCS-IRES-VENUS (kindly provided by Dr. Hiroyuki Miyoshi, RIKEN BioResource Center, Ibaraki, Japan) containing the coding region of *LSD1* cDNA for gain-of-function experiments. We used the lentiviral short-hairpin RNA/short-interfering RNA (shRNA/siRNA) expression vector pLL3.7 for knockdown experiments. Oligonucleotides containing siRNA target sequences are as follows: sense, 5’-TTGAATTAGC TGAAACACAA TTCAAGAGA ttgtgtttcagctaattca TTTTTTC-3’, antisense, 5’-TCGAGAAAAA ATGAATTAGC TGAAACACAA TCTCTTGAA ttgtgtttcagctaattca A-3’. Scrambled sequences were used as controls. These vectors were co-transfected into 293FT cells with packaging plasmids (Invitrogen) to produce infective lentiviruses in culture supernatants. Lentiviruses were then added to cell suspensions in the presence of 8 μg/ml polybrene and transduced for 24 hours as previously described [33].

### Teratoma formation assay

Approximately 2×10^6^ hiPSCs were collected, washed with PBS and resuspended in 200 μl diluted (1:1) Matrigel solution (Becton Dickinson). Cells were inoculated subcutaneously into non-obese diabetic/severe combined immunodeficiency (NOD/SCID) mice (Charles River Laboratories, Wilmington, MA). The developed teratomas were excised 3–7 weeks after inoculation. After sectioning, slides containing various regions of teratomas were stained by hematoxyline-eosin (HE) and analyzed under a microscope. For *ex vivo* tracing of teratomas, we established luciferase-expressing sublines of ChiPS17, designated ChiPS17-Luc, by transfecting firefly luciferase cDNA [34]. ChiPS17-Luc cells were inoculated subcutaneously in NOD/SCID mice. Drugs were administered intraperitoneally in 200 μL volume of solution containing 3% DMSO and 97% sterile 0.9% NaCl. The control group received the vehicle (3% DMSO in 0.9% NaCl) alone on the same schedule. Tumor burden was monitored by measuring teratoma-derived luciferase activity with the noninvasive bioimaging system. In short, tumor-bearing mice were injected with 1.5 mg of the luciferase substrate D-luciferin (Promega, Fitchburg, WI) intraperitoneally after being anesthetized with isoflurane. Photons transmitted through the body were collected for a specified length of time and analyzed using the IVIS Imaging System with Living Image software (Xenogen, Alameda, CA). Quantitative data were expressed in units of photons (photons/s) [34].

### Drugs

The drugs used in this study were tubastatin A (Enzo Life Science, Am Arbor, MI), panobinostat, bortezomib, OG-L002, GSK2879552 (Selleck Chemicals, Houston, TX), and S2101 (Millipore, Temecula, CA). S2116 and S2157 were synthesized by Tokyo Chemical Industry Co., Ltd. (TCI), and the syntheses will be described elsewhere (Niwa et al., manuscript in preparation). All drugs were dissolved in dimethyl sulfoxide at appropriate concentrations. A pharmacokinetic analysis was performed following a single intraperitoneal (i.p.) dose of S2157 (50 mg/kg), and serial blood samples were collected at 0.5, 1, 2, 4 and 8 hrs after administration. Blood samples were centrifuged, and the plasma samples were deproteinized with acetonitrile. The supernatants were subjected to LC-MS/MS analysis to determine the plasma S2157 concentrations.

### Inhibition assay

IC_50_ values of LSD1 demethylase inhibition were obtained by the peroxidase-coupled reaction method as described previously [35, 36]. Briefly, 1 μM human LSD1 was incubated with serial dilutions of inhibitors in 50 mM HEPES-Na (pH 7.5) buffer containing 400 μM 4-aminoantipyrine, modified Trinder’s reagent TOOS (N-ethyl-N-(2-hydroxy-3-sulfopropyl)-3-methylaniline, sodium salt dihydrate), and 40 μg/ml horseradish peroxidase at 25°C for 10 min. The reaction mixture was subsequently incubated with 100 μM K4-dimethylated H3 tail peptide (1–20) for 30 min. Absorption of the peroxidase by-product generated by lysine demethylation was measured at 562 nm with a 96-well microplate reader (Ultrospec Visible Plate Reader II 96; GE Healthcare). IC_50_ values were calculated with Prism 6 software (version 6.0e), using dose-response results in triplicate. LSD2 inhibition assays were performed using 200 μM K4-dimethylated H3 tail peptide (1–20) and 1 μM human LSD2. MAO inhibition assays were performed using 50 or 150 μM tyramine, 100 μg/ml MAO-A (Sigma-Aldrich) and 200 μg/ml MAO-B (Sigma-Aldrich).

### Cell proliferation assays

Cell proliferation was monitored using Cell Counting Kit (Wako Biochemicals). Cells were seeded in 96-well flat-bottomed microplates. Absorbance was measured at a wavelength of 450 nm using a microplate reader and expressed as a percentage of the value of the corresponding untreated cells.

### Statistics

We used one-way ANOVA with Tukey’s multiple comparison test and Student’s *t* test to determine statistical significance. *P* values less than 0.05 were considered significant.

## SUPPLEMENTARY MATERIALS FIGURES


