# Supplementary material for: Lysine-specific demethylase 1 inhibitors prevent teratoma development from human induced pluripotent stem cells
Source: Oncotarget. 2018 Jan 8;9(5):6450–62. doi: 10.18632/oncotarget.24030 (PMC5814224; doi:10.18632/oncotarget.24030)
Supplement: Supplementary file 1 [file oncotarget-09-6450-s001.pdf]

Lysine-specific demethylase 1 inhibitors prevent teratoma development from human induced pluripotent stem cells

SUPPLEMENTARY MATERIALS

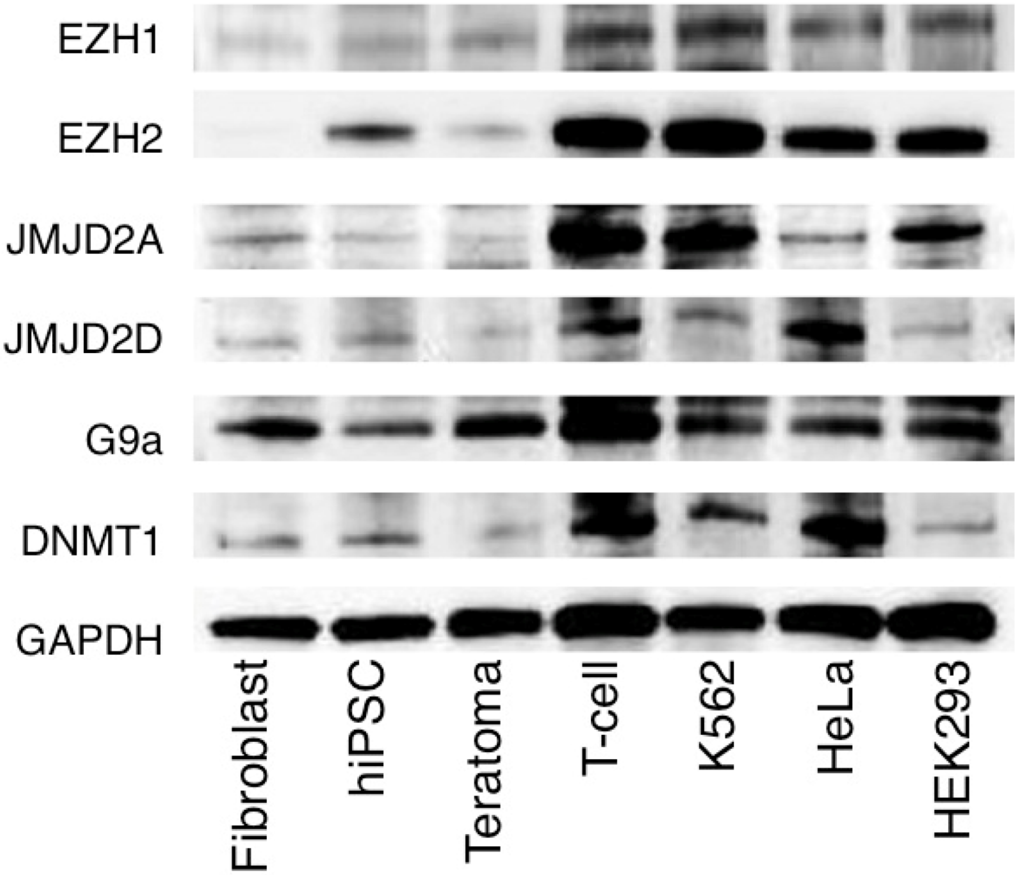

Supplementary Figure 1: We isolated whole cell lysates from normal human fibroblasts, ChiPS17, ChiPS17-derived teratoma, normal human T-lymphocytes, and cancer cell lines (K562, HeLa, and HEK293) for immunoblot analyses to determine the expression of EZH1, EZH2, JMJD2A, JMJD2D, G9a, DNMT1, and GAPDH (internal control).

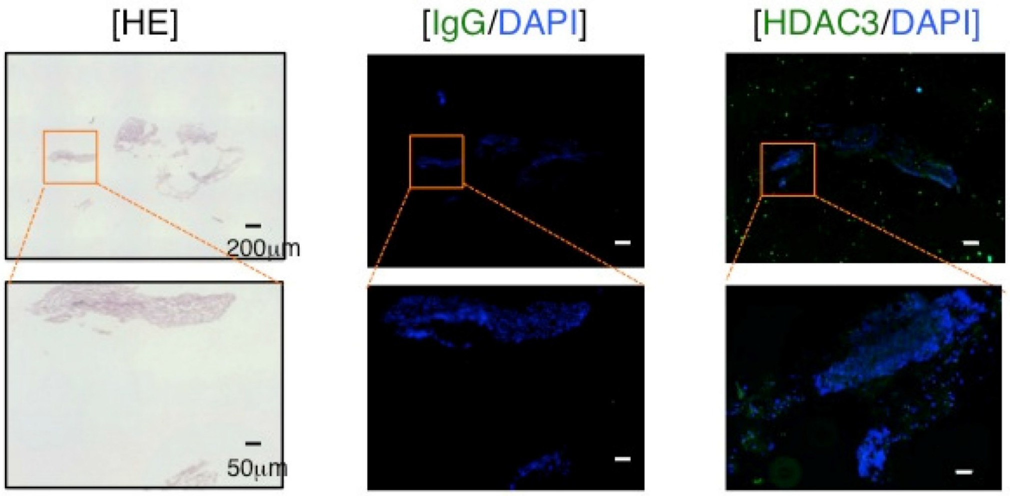

Supplementary Figure 2: Frozen continuous sections were prepared from the developed teratomas and subjected to hematoxylin-eosin (HE) and immunofluorescent chemical (IFC) staining. IFC specimens were stained with anti-HDAC3 antibody or isotype-matched control (IgG), followed by staining with Alexa Fluor 488-conjugated anti-rabbit IgG (green). Nuclei were counterstained with DAPI (blue). Only merged images are shown. Scale bars indicate 200 µm (upper panels) and 50 µm (lower panels), respectively. Data shown are representative of multiple independent experiments.

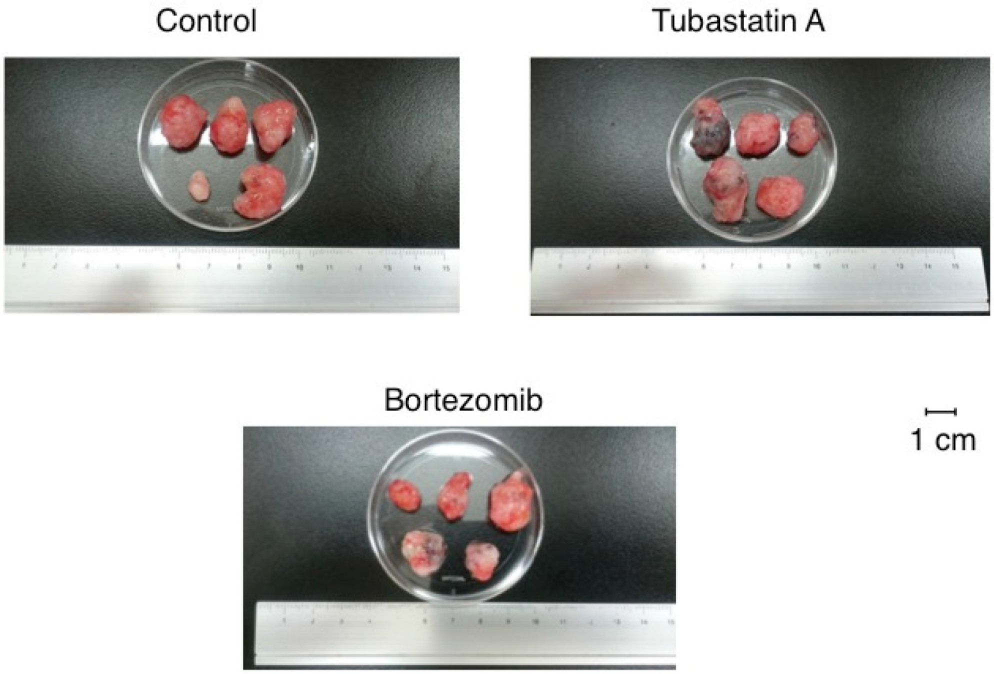

Supplementary Figure 3: We subcutaneously inoculated 2 × 10^6 ChiPS17 cells into the right thigh of NOD/SCID mice. Immediately after transplantation, mice were randomly divided into three groups and intraperitoneally administered 50 mg/kg tubastatin A (n=5), 0.5 mg/kg bortezomib (n=5), or vehicle control (0.9% NaCl) (n=5) twice a week for 5 weeks. Shown are representative photographs of teratomas on day 56 (original magnification: ×2).

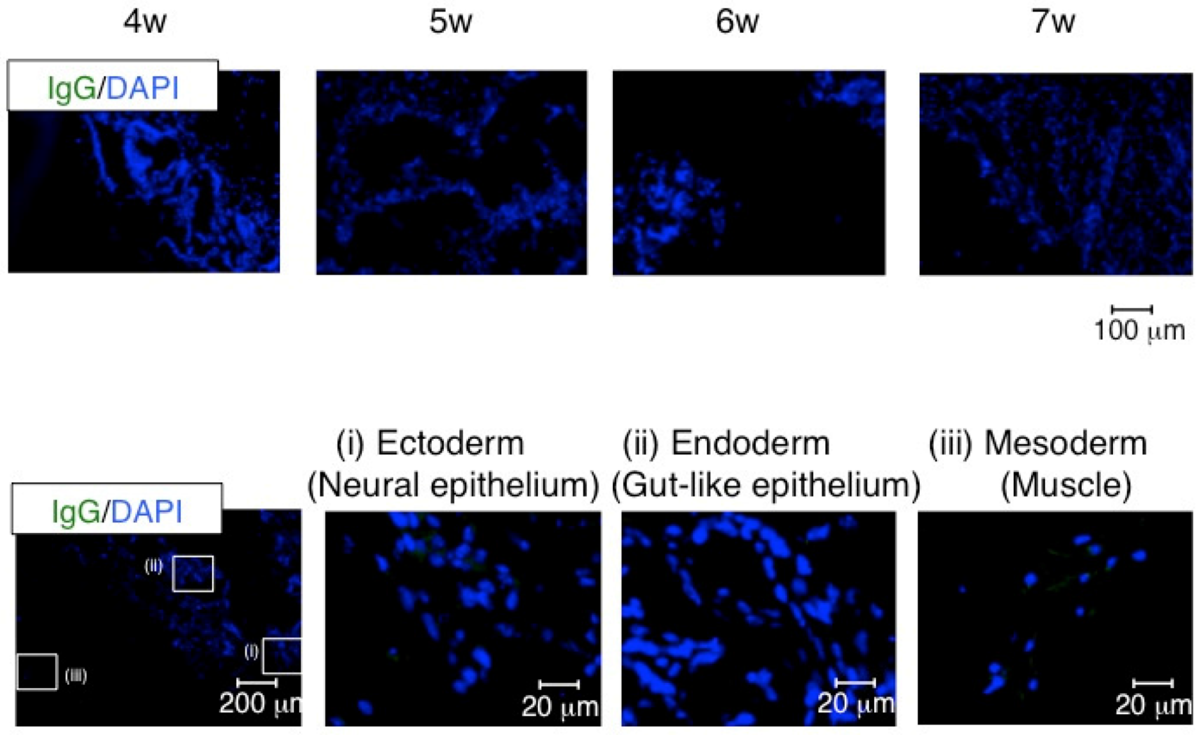

Supplementary Figure 4: Frozen continuous sections were prepared from the developed teratomas and subjected to IFC staining. The specimens were stained with unimmunized rabbit IgG, followed by staining with Alexa Fluor 488-conjugated anti-rabbit IgG antibody (green). Nuclei were counterstained with DAPI (blue). Only merged images are shown. Scale bars indicate 100 µm (upper panels), 200 µm (lower left end panel), or 20 µm (lower right 3 panels). Data shown are representative of multiple independent experiments.

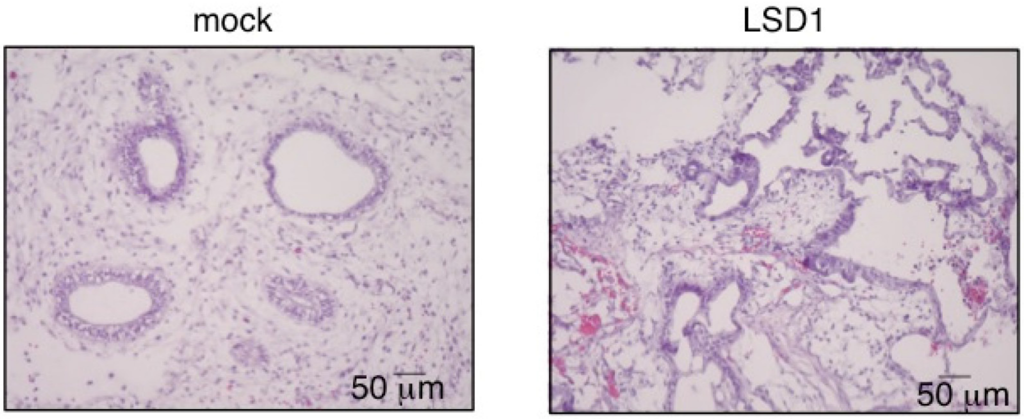

Supplementary Figure 5: NOD/SCID mice were inoculated subcutaneously with 2 × 10^6 cells of ChiPS17-mock or ChiPS17-LSD1-H12 into the right thigh. Frozen sections were prepared from the developed teratomas and subjected to HE staining. Scale bars indicate 50 μm. Data shown are representative of multiple independent experiments.

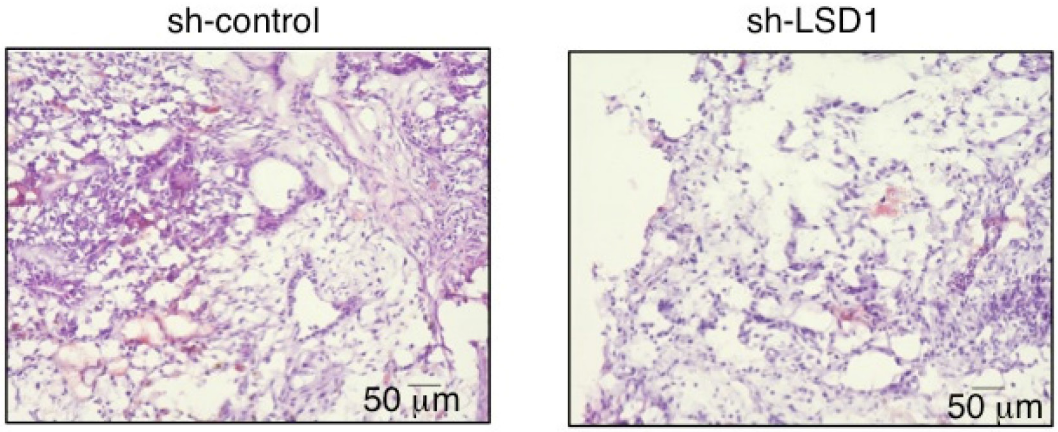

Supplementary Figure 6: NOD/SCID mice were inoculated subcutaneously with 1 × 10^6 cells of ChiPS17-sh-control or ChiPS17-sh-LSD1-A2 into the right thigh. Frozen sections were prepared from the developed teratomas and subjected to HE staining. Scale bars indicate 50 μm. Data shown are representative of multiple independent experiments.

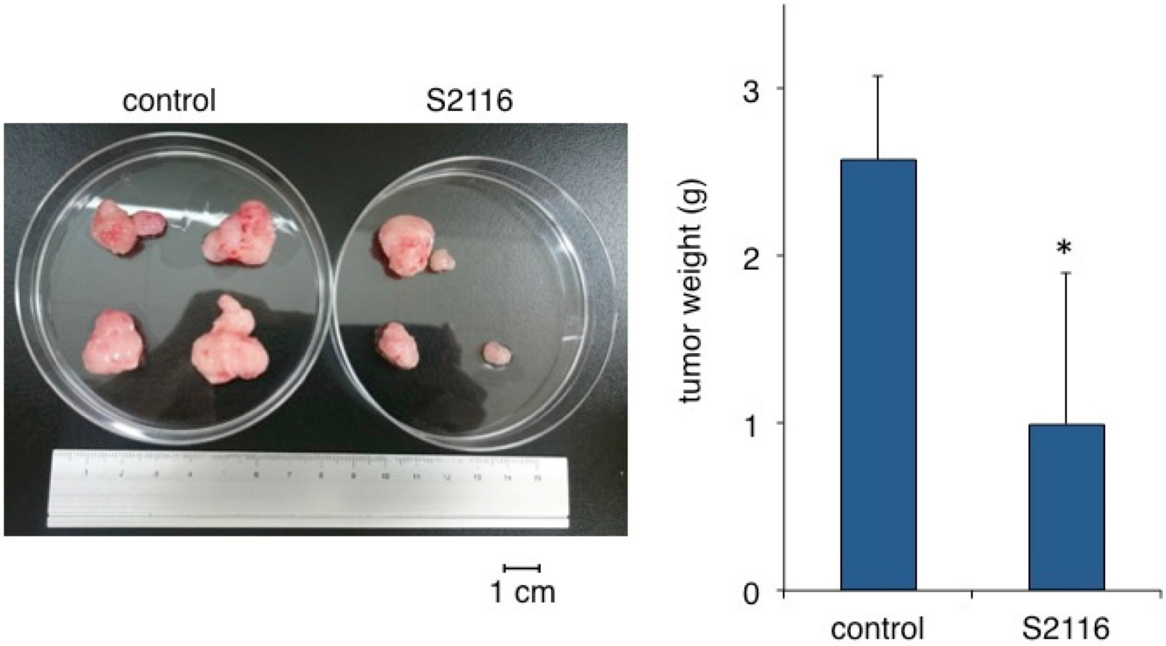

Supplementary Figure 7: We subcutaneously inoculated 2 × 10^6 201B cells into the right thigh of NOD/SCID mice. Immediately after transplantation, mice were intraperitoneally administered 30 mg/kg S2116 (n=3) or vehicle (0.9% NaCl) (n=4) five times per week for three weeks. Left panel: Shown are representative photographs of teratomas on day 80 (original magnification: ×2). Right panel: The y-axis shows the average tumor weight of the developed teratomas. Bars depict means ± S.D. $^{*}P$ <0.05 determined using Student's *t* test.
